# Impact of Empiric Antimicrobial Therapy on Outcomes in Patients with *Escherichia coli *and *Klebsiella pneumoniae *Bacteremia: A Cohort Study

**DOI:** 10.1186/1471-2334-8-116

**Published:** 2008-09-15

**Authors:** Kerri A Thom, Marin L Schweizer, Regina B Osih, Jessina C McGregor, Jon P Furuno, Eli N Perencevich, Anthony D Harris

**Affiliations:** 1 Department of Epidemiology and Preventive Medicine, University of Maryland School of Medicine, Baltimore, MD, USA; 2 Department of Medicine, Centre Hospitalier Universitaire Vaudois and University of Lausanne, Lausanne, Switzerland; 3 College of Pharmacy, Oregon State University, Portland, OR, USA; 4 Vetrans Affairs Maryland Health Care System, Baltimore, MD, USA

## Abstract

**Background:**

It is unclear whether appropriate empiric antimicrobial therapy improves outcomes in patients with bacteremia due to *Escherichia coli *or *Klebsiella*. The objective of this study is to assess the impact of appropriate empiric antimicrobial therapy on in-hospital mortality and post-infection length of stay in patients with *Escherichia coli *or *Klebsiella *bacteremia while adjusting for important confounding variables.

**Methods:**

We performed a retrospective cohort study of adult patients with a positive blood culture for *E. coli *or *Klebsiella *between January 1, 2001 and June 8, 2005 and compared in-hospital mortality and post-infection length of stay between subjects who received appropriate and inappropriate empiric antimicrobial therapy. Empiric therapy was defined as the receipt of an antimicrobial agent between 8 hours before and 24 hours after the index blood culture was drawn and was considered appropriate if it included antimicrobials to which the specific isolate displayed *in vitro *susceptibility. Data were collected electronically and through chart review. Survival analysis was used to statistically assess the association between empiric antimicrobial therapy and outcome (mortality or length of stay). Multivariable Cox proportional hazards models were used to calculate hazard ratios (HR) and 95% confidence intervals (CI).

**Results:**

Among 416 episodes of bacteremia, 305 (73.3%) patients received appropriate empiric antimicrobial therapy. Seventy-one (17%) patients died before discharge from the hospital. The receipt of appropriate antimicrobial agents was more common in hospital survivors than in those who died (p = 0.04). After controlling for confounding variables, there was no association between the receipt of appropriate empiric antimicrobial therapy and in-hospital mortality (HR, 1.03; 95% CI, 0.60 to 1.78). The median post-infection length of stay was 7 days. The receipt of appropriate antimicrobial agents was not associated with shortened post-infection length of stay, even after controlling for confounding (HR, 1.11; 95% CI 0.86 to 1.44).

**Conclusion:**

Appropriate empiric antimicrobial therapy for *E. coli *and *Klebsiella *bacteremia is not associated with lower in-hospital mortality or shortened post-infection length of stay. This suggests that the choice of empiric antimicrobial agents may not improve outcomes and also provides data to support a randomized trial to test the hypothesis that use (and overuse) of broad-spectrum antibiotics prior to the availability of culture results is not warranted.

## Background

*Escherichia coli *and *Klebsiella pneumoniae *are the leading causes of Gram-negative bloodstream infections in the U.S. and Canada[1]. The overall mortality associated with *E. coli *bacteremia may be as high as 20% [2-4], while the mortality for *K. pneumoniae *bacteremia has been estimated to range from 19–50%[2,4-6]. In addition, Gram-negative bacilli are becoming increasingly resistant to antimicrobials. The National Nosocomial Infections Surveillance (NNIS) System reported a 47% increase in nosocomial infections due to third-generation cephalosporin-resistant *K. pneumoniae *in 2003 compared to the previous five years[7]. Increasing antimicrobial resistance of these organisms may decrease the likelihood of receiving appropriate empiric antimicrobial therapy.

Pathogen-directed, "appropriate" antimicrobial therapy is considered fundamental in the treatment of bacteremia. Early in the course of infection, however, the causative organism is typically unknown and the choice of therapy is empiric, thus clinicians often choose broad-spectrum antimicrobials to cover a wide range of potential pathogens. This strategy may result in increases in cost, adverse events and antimicrobial resistance and therefore, it is important to determine if there is a true association with patient outcome. Research to date, however, has not consistently demonstrated an association between receipt of appropriate empiric antimicrobial therapy and improved outcomes in patients with bacteremia due to *E. coli *or *Klebsiella *species [8-19].

Most studies which have evaluated the effect of empiric antimicrobial therapy on patient outcomes included bacteremia due to all bloodstream pathogens or to Gram-negative organisms in general, thus decreasing their statistical power to evaluate outcomes specific to *E. coli *or *Klebsiella *bacteremias. To our knowledge, five studies have exclusively examined the association between empiric antimicrobial therapy and outcome among patients with *E. coli *or *Klebsiella *bacteremia[8,9,15,18,19]. These studies were limited in their generalizability either by small samples or by including only episodes of bacteremia due to extended-spectrum beta-lactamase producing strains[8,9,15]. In addition, while most studies controlled for severity of illness by using some aggregate score, at least two of these studies controlled for severity of illness after the onset of bacteremia[18,19] while two other studies did not clearly state when this variable was measured[9,15]. We argue that measurement of severity of illness at the time of bacteremia or later may reflect complications of the current infection and not the patient's underlying disease status. Therefore, aggregate scores used to approximate severity of illness should be measured at some time point prior to the onset of bacteremia. In this study, we aimed to evaluate the effect of empiric therapy on in-hospital mortality and post-infection length of stay among adult patients with bacteremia due to *E. coli *or *Klebsiella *species, while controlling for severity of illness before the detection of bacteremia.

## Methods

### Study Population

This study was performed at the University of Maryland Medical Center (UMMC), a 656-bed tertiary-care hospital located in Baltimore, Maryland. The UMMC provides specialized medical and surgical care, including bone marrow and solid organ transplantation. All adult patients (> 18 years of age) with a positive blood culture for either *E. coli *or *Klebsiella *species between January 1, 2001 and June 8, 2005 were eligible for inclusion. Patients were identified using the UMMC central data repository, a relational database that contains patient clinical and administrative data. Patients with multiple episodes of bacteremia during the study period were allowed to enter the cohort multiple times only if the episodes of bacteremia occurred during separate hospital admissions. Patients with a polymicrobial bacteremia were included in this study. However, cases in which the index blood culture was positive for both *E. coli *and *Klebsiella *species were only included once.

### Study Design

We used a retrospective cohort design to evaluate the effects of empiric antimicrobial therapy on morbidity and mortality associated with *E. coli *and *Klebsiella *bacteremia. The primary outcome measure, in-hospital mortality, was compared among subjects who received appropriate and inappropriate empiric therapy. We assessed post-infection length of stay (the time period from the date the index blood culture was obtained to the date of discharge) as the secondary outcome measure. This study was approved by the University of Maryland Institutional Review Board and was deemed exempt from informed consent.

### Variable definitions

Empiric antimicrobial therapy was defined as the receipt of an antimicrobial agent by the patient between 8 hours before and 24 hours after the index blood culture was drawn. We chose this early time period in attempt to exclude scenarios where preliminary microbiologic data were available (e.g. knowledge of whether the organism was a lactose-fermenter) since this may have influenced antimicrobial choice. Empiric therapy was considered appropriate if it included intravenous and/or oral antimicrobials to which the specific isolate (or isolates, if polymicrobial) displayed *in vitro *susceptibility.

Severity of illness prior to bacteremia was assessed by calculating a modified Acute Physiology Score (APS) based on the Acute Physiology and Chronic Health Evaluation (APACHE) III score 24 hours before the time the index culture was obtained [20-22]. We chose this time point to better ensure that the aggregate score accurately reflected the baseline severity of illness of each patient and did not include values that occurred as a consequence of the bacteremia [23]. If the index blood culture was obtained within 24 hours of hospital admission then the modified APS was calculated at admission. The APACHE III score was designed for use among intensive care unit (ICU) patients. Since this study included participants who many not have been in an ICU at the time of bacteremia, we modified the score by excluding variables that were not applicable to our study population (i.e. pulmonary arterial gradient, urine output, neurologic status and ventilator data) as has been done in other studies [20-22,24].

The presence of pre-existing comorbid conditions was determined using the Chronic Disease Score (CDS), an aggregate comorbidity measure which utilizes patient medications as indicators for the presence of comorbid conditions[25]. In this study, as has been done in other studies, the CDS was calculated based upon the medications ordered within the first 24 hours of hospital admission [20,26,27].

### Data Collection

The central data repository was used to collect administrative, pharmacy, laboratory and outcome data for all patients. The data contained within the tables of the repository have been validated against medical records for this and previous research studies and have positive and negative predictive values of greater than 98%[20,27]. Variables electronically collected included demographics, date and time the blood culture of interest was obtained, time at risk (i.e. time from hospital admission to collection of index blood culture), post-infection length of stay, time to susceptibility results (i.e. time from collection of index blood culture to receipt of antimicrobial susceptibility testing results), antimicrobial susceptibility results for the organism, laboratory data included in the APACHE III score and medication data used to calculate the CDS. For polymicrobial bacteremias, all co-infecting species were identified and the antimicrobial susceptibility profile was reviewed for all organisms.

Patient medical records were used to collect additional information such as vital signs and the presence of a central venous catheter at time of culture collection. Medication administration records were examined for each patient to determine if and when the patient received empiric antimicrobial therapy.

### Statistical analysis

All data were analyzed using SAS software version 9.1 (SAS Institute, Cary, NC). The Fisher's exact test and Chi-square test were used to compare categorical variables and the Student's t-test was used for continuous variables.

Data for both outcomes were analyzed and compared between the two groups (appropriate and inappropriate empiric therapy) using Kaplan-Meier survival models. The log-rank and Wilcoxon tests were used to compare survival curves. Multivariable survival analysis, using the Cox proportional hazards model, was done to assess the association between empiric antimicrobial therapy and the outcomes, in-hospital mortality and length of stay, while adjusting for potential confounding factors. Variables that were significantly associated (p < 0.05) with the outcome or that modified the regression model coefficient for empiric therapy by more than 10% were included in the final multivariable model. The exposure, appropriate therapy, was forced into the model regardless of statistical significance. For the outcome of length of stay, the event was discharge from hospital and therefore, the term survival refers to patients remaining in the hospital (i.e., those who "survived" from being discharged). Patients who died in the hospital were excluded from this analysis because their hospital stay was shortened by death. All tests of significance were two-tailed, and p-values of less than 0.05 were considered significant. Hazards ratios (HRs) and 95% confidence intervals (CIs) were calculated. We examined the associations between appropriate empiric therapy and outcomes for all observations, and then again after stratification by organism.

## Results

During the study period, 416 episodes of *E. coli *and *Klebsiella *bacteremia were included. Two-hundred and twenty-five (54.0%) bacteremias were due to *E. coli *and 203 (48.8%) were due to *Klebsiella *species (175 *K. pneumoniae*, 28 *K. oxytoca*, 2 non-speciated *Klebsiella *isolates). Two hundred and three (48.8%) of the positive cultures were obtained at or after 48 hours following admission (i.e. hospital-acquired) and the remaining 213 (51.2%) were obtained within the first 48 hours of admission (i.e. community-acquired). Eighty-nine (21.4%) of the bacteremia episodes were polymicrobial and 11 of these included both *E. coli *and *K. pneumoniae*. The characteristics of this cohort are presented in Table 1.

**Table 1:** Baseline characteristics of the study population

Variable	Entire Cohort (N = 416)	*E. coli* (N = 225)*	*Klebsiella* (N = 203)*
Age (mean in years, SD)	55.3 ± 16.2	55.7 ± 16.2	54.9 ± 15.8
Male sex	45.3% (188/415)	49.1% (110/224)	40.9% (83/203)
Hospital-acquired Bacteremia^†^	48.8% (203/416)	39.1% (88/225)	59.6% (121/203)
Polymicrobial bacteremia	21.4% (89/416)	20.9% (47/225)	26.6% (54/203)
Appropriate empiric antimicrobial therapy	73.3% (305/416)	74.7% (168/225)	70.9% (144/203)
Admission to the ICU	37% (154/416)	36.0% (81/225)	39.4% (80/203)
Mechanical ventilation at admission	9.6% (40/415)	6.7% (15/224)	13.3% (27/230)
Mechanical ventilation at culture	16.6% (69/415)	14.7% (33/224)	19.7% (40/203)
Presence of central line at culture	54.4% (225/414)	43.5% (97/223)	68.5% (139/203)
APS before culture (mean, SD)	22.7 ± 14.2	22.5 ± 14.6	22.9 ± 13.7
Chronic disease score (mean, SD)	7.1 ± 3.9	6.9 ± 4.0	7.4 ± 3.7
Time at risk (median in days, IQR)^††^	1.5 (0.1 to 10.0)	0.4 (0.1 to 6.9)	5.1 (0.2 to 13.3)
Time to susceptibility (median in days, IQR)^†††^	3.1 (2.6 to 3.9)	3.1 (2.6 to 3.9)	3.2 (2.6 to 4.1)
Length of stay (median in days, IQR)^††††^	7.0 (3.9 to 14.2)	6.2 (3.7 to 12.1)	8.7 (4.3 to 17.6)
In-hospital mortality	17.0% (71/416)	17.8% (40/225)	18.2% (37/203)

ICU – Intensive care unit

SD – standard deviation

APS – modified acute physiology score

IQR – interquartile range

* Eleven patients had polymicrobial bacteremia which included both *E. coli *and *Klebsiella *species

^†^Bacteremia which occurred at or greater than 48 hours after hospital admission

^††^Time at risk is the time from hospital admission to index culture collection

^†††^Time to susceptibility is the time from index culture collection to the receipt of antibiotic susceptibility testing results

^††††^Length of stay is the time from index culture collection to hospital discharge or death

Three-hundred and five (73.3%) patients received appropriate empiric antimicrobials according to our study definition. For the antimicrobial sensitivity patterns of the isolates please see Additional File 1: Antimicrobial Susceptibilities of *Escherichia coli *and *Klebsiella *Bloodstream Isolates. The median time (interquartile range, IQR) between culture collection and receipt of antimicrobial susceptibility testing results was 3.1 days (2.6 to 3.9 days).

The mean modified APS 24 hours before culture collection was 22.7 (SD = 14.2). For 199 (48%) episodes of bacteremia, the index blood culture was drawn within 24 hours of hospital admission and therefore the components used to calculate the APS values used were measured at the time of hospital admission.

### In-hospital mortality

Seventy-one (17.0%) patients died before discharge from the hospital. In-hospital mortality among patients with *E. coli *bacteremia was 17.8% (40/225) and among patients with *Klebsiella *bacteremia was 18.2% (37/203). Table 2 displays characteristics of hospital survivors and non-survivors. Factors significantly associated with in-hospital mortality included: older age, polymicrobial bacteremia, presence of a central venous catheter at the time of culture, higher baseline severity of illness and shorter time at risk. In addition, the receipt of empiric appropriate antimicrobial therapy was more common among hospital survivors than those who died (p = 0.04).

**Table 2:** Predictors for hospital mortality in patients with bacteremia due to *E. coli *or *Klebsiella*

Variable	Hospital Non-survivors n = 71 (17%)	Hospital Survivors n = 345 (83%)	P-value
Age (mean in years, SD)	59.6 ± 14.6	54.4 ± 16.3	0.01
Male sex (n, %)	29(40.9)	159(46.2)	0.41
Polymicrobial bacteremia (n, %)	22(31.0)	67(19.4)	0.03
Appropriate empiric antimicrobial therapy (n, %)	45(63.4)	260(75.4)	0.04
Presence of central line at time of culture (n, %)	48(67.6)	177 (51.6)	0.01
APS before culture (mean, SD)	34.9 ± 14.8	20.2 ± 13.8	< 0.01
Chronic disease score (mean, SD)	7.9 ± 3.9	7.0 ± 3.9	0.08
Time at risk (median in days, IQR)^†^	8.0 (0.17 to 21.4)	0.9 (0.1 to 8.8)	< 0.01
Time to susceptibility (median in days, IQR)^††^	3.2 (2.8 to 4.1)	3.0 (2.6 to 3.9)	0.02

APS – modified acute physiology score

IQR – interquartile range

^†^Time at risk is the time from hospital admission to index culture collection

^††^Time to susceptibility is the time from index culture collection to the receipt of antibiotic susceptibility testing results

P-values were calculated using the Fisher's exact test or Chi-squared tests for categorical variables and the Student's t-test for continuous variables.

**Figure 1:**
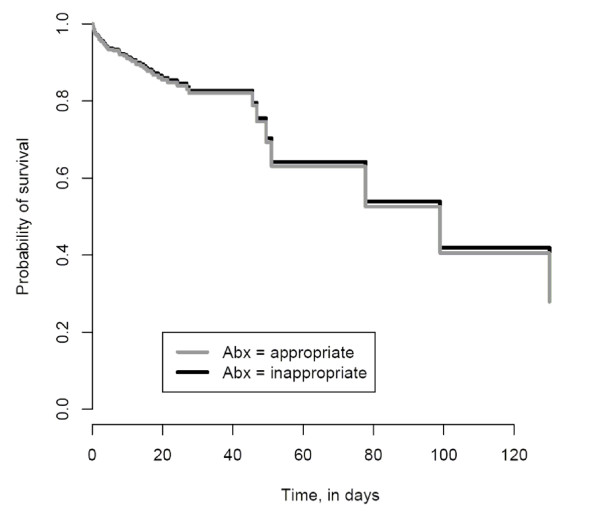
**In-Hospital Survival Curve based on Cox Proportional Hazards Models**. In-hospital survival among patients who received appropriate (grey line) and inappropriate empiric therapy (black line).

After controlling for confounding variables (age, severity of illness, the presence of a central venous catheter at the time of culture), there was no association between the receipt of appropriate empiric antimicrobial therapy and mortality (HR, 1.03; 95% CI, 0.60 to 1.78)(Table 3). Figure 1 shows the survival curves for patients with bacteremia who received appropriate and inappropriate empiric antimicrobial therapy based on the Cox proportional hazards models. Multivariable survival analysis suggested that severity of illness (HR, 1.06; 95%CI, 1.05 to 1.08) and age (HR, 1.02; 95% CI, 1.01 to 1.04) were independent predictors of in-hospital mortality. Similar analyses were performed separately for *E. coli *and *Klebsiella *bacteremia (Table 3).

**Table 3:** Multivariable Cox Proportional Hazards Models

	Outcome = In-hospital Mortality	Outcome = Length of Stay
Variable	Hazard Ratio (95% CI)	Hazard Ratio (95% CI)

All Bacteremias (N = 416)		
Appropriate Antibiotics -8 to 24 hours	1.03 (0.60 to 1.78)	1.11 (0.86 to 1.44)
Age (per year)	1.02 (1.01 to 1.04)	NS
Male Sex	--	NS
Central line at time of culture	--	0.67 (0.54 to 0.85)
Time at risk^† ^(per day)	NS	0.66 (0.52 to 0.83)
APS 24 hours before culture	1.06 (1.05 to 1.08)	0.99 (0.97 to 0.99)
Polymicrobial bacteremia	NS	--
		
E. coli Bacteremia (N = 225)		
Appropriate Antibiotics -8 to 24 hours	1.11 (0.52 to 2.34)	1.04 (0.72 to 1.51)
Central line at time of culture	NS	NS
Time at risk^†^	NS	0.66 (0.47 to 0.91)
APS 24 hours before culture	1.06 (1.04 to 1.08)	0.98 (0.97 to 1.00)
Polymicrobial bacteremia	NS	--
		
Klebsiella Bacteremia (N = 203)		
Appropriate Antibiotics -8 to 24 hours	0.84 (0.42 to 1.70)	1.21 (0.84 to 1.73)
Age	1.03 (1.01 to 1.06)	--
Time at risk^†^	--	0.65 (0.47 to 0.90)
Central line at time of culture	--	0.62 (0.44 to 0.86)
APS 24 hours before culture	1.06 (1.03 to 1.09)	0.98 (0.97 to 1.00)

APS – modified acute physiology score

NS – not significant (p > 0.05)

-- Not included in final model

^†^Time at risk is the time from hospital admission to index culture collection

NB: Hazard Ratios are reported per one unit increase in the study variable; for example a HR of 1.06 for APS can be interpreted as a 6% increase in the hazard of death for each one unit increase in APS; therefore for an increase in APS of 5 points the hazard of death would increase by 30%.

### Length of stay

The median post-infection length of stay in the hospital after collection of the index blood culture was 7 days (IQR, 3.9 to 14.2 days). The median post-infection length of stay among patients with *E. coli *bacteremia was 6.2 days (IQR, 3.7 to 12.1 days) and among patients with *Klebsiella *bacteremia was 8.7 days (IQR, 4.3 to 17.6 days). In bivariable analyses, male sex (p = 0.04), presence of a central venous catheter at the time of culture (p < 0.01), severity of illness (p < 0.01), CDS (p < 0.01), and time at risk (p < 0.01) were statistically associated with an increased post-infection length of stay. However, the receipt of appropriate antimicrobial agents within 24 hours of the time the index blood culture was obtained was not associated with post-infection length of stay (p = 0.24).

**Figure 2:**
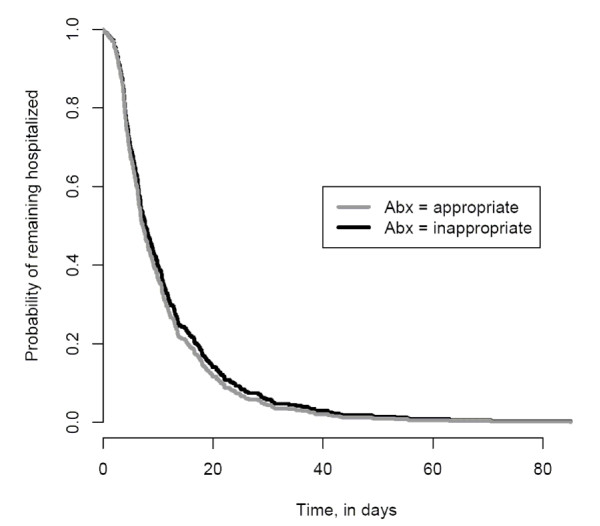
**Time to Discharge Curve based on Cox Proportional Hazards Models**. Time to discharge after index blood culture among patients who received appropriate (grey line) and inappropriate empiric antibiotics (black line).

After controlling for confounding variables (the presence of a central venous catheter at the time of culture, severity of illness and time at risk), appropriate empiric antimicrobial therapy was not associated with time to discharge from the hospital (HR, 1.11; 95% CI, 0.86 to 1.44, Table 3). Figure 2 shows the time to discharge curves for patients who received appropriate and inappropriate empiric antimicrobial therapy based on the Cox proportional hazard models. For this curve the "event" is hospital discharge and the term "survival" refers to patients who remain in the hospital. Multivariable survival analysis demonstrated that severity of illness (HR, 0.99; 95% CI, 0.97 to 0.99), the presence of a central venous catheter at the time of culture (HR, 0.67; 95% CI, 0.54 to 0.85) and the time at risk (HR, 0.66; 95% CI, 0.52 to 0.83) were independent predictors of time to discharge (i.e. were associated with an increased length of stay).

## Discussion

We evaluated the effect of empiric antimicrobial therapy on outcomes among a cohort of patients with *E. coli *and *Klebsiella *bacteremia and did not observe a significant association between appropriate empiric antimicrobial therapy and in-hospital mortality or post-infection length of stay, after controlling for important confounders such as severity of illness.

Though it may seem logical that the use of appropriate antimicrobials early in the course of therapy for *E. coli *or *Klebsiella *bacteremia would lead to improved outcomes; this association has not been clearly established. Differences in methodologies among previous studies may explain why some, but not all studies have observed significant associations between appropriate therapy and clinical outcomes. Previously published studies have varied by infecting organism, patient population, classification of appropriate therapy and the time this variable was measured, whether or not severity of illness was controlled for in the final analysis, and the time at which severity of illness was measured.

Empiric therapy is considered to be the receipt of antimicrobials during the time period prior to the availability of bacterial culture and susceptibility testing results. The empiric therapy time intervals vary in the literature, from within 24 hours of the bacterial culture results to the time antimicrobial susceptibility testing results are available[28]. In some instances, however, preliminary results may be available to physicians prior to definitive susceptibility results which could influence antibiotic choice and thus bias the analyses from finding a beneficial effect of appropriate empiric therapy. For this reason, we chose to evaluate empiric therapy early in the course of the disease process, from 8 hours prior to the time in which the index culture was obtained to 24 hours afterwards.

Underlying severity of illness is an important predictor of mortality. Therefore, physicians may be more likely to prescribe broad-spectrum empiric antimicrobial therapy for presumed bacteremia in patients who have more severe illness at baseline[16]. Due to the established association with mortality and the putative association with antimicrobial therapy, baseline severity of illness is a potential confounding factor of the association between these two variables. As a result, baseline severity of illness should be controlled for in any analysis comparing antimicrobial therapy and outcomes in patients with bacteremia. Several studies have measured severity of illness at the time of bacteremia or later. However, some components of the APS measured in this time period are likely to be affected by the infection and may not reflect the baseline severity of illness and thus should not be controlled for[23]. The optimal time to measure severity of illness is just prior to the onset of bacteremia, but this time period is difficult to identify. In this study we measured the severity of illness using the modified APS 24 hours before the index culture was obtained. Yet, for some observations the index blood culture was obtained within 24 hours of admission to the hospital and in these instances the modified APS was calculated using variables collected at the time of admission. In order to evaluate the impact of different measurement times, we performed a secondary analysis on the 217 observations for which the modified APS was calculated at or greater than 24 hours prior to the time the index blood culture was obtained and obtained results similar to those presented for the entire cohort (data not shown).

A limitation of this study is that the design is observational, and therefore it is impossible to completely control for all variables which may be associated with both mortality and the receipt of empiric antibiotics. Also, because this study was retrospective and observational, antimicrobial choice was made solely by physicians (there are no specific hospital-based protocols for the use of antimicrobial agents at this institution). We were unable to collect data on other potential factors which may have influenced the physician's choice of empiric therapy, such as individual prescribing patterns and physician knowledge of a patient's previous infections, previous antimicrobial susceptibility data or prior antibiotic use. Additionally, dosing of antimicrobial agents was not considered and therefore it is possible that selection of appropriate antimicrobials may lead to clinical failure if the antimicrobial agent was not adequately dosed. Furthermore, due to frequent changes in antimicrobial agents and inadequate power we were unable to consider the specific antimicrobial agent received in the analysis. Another potential limitation in this study is the use of a modified APS score to measure severity of illness in non-critically ill patients. While this score has not been validated specifically for this purpose, it has been used extensively in the infectious disease literature[5,9,20,29]. In addition, we did not have data on patients after discharge from the hospital and therefore we were limited to evaluating in-hospital morality as our outcome instead of overall mortality. Finally, we did not evaluate the source of bacteremia, nor source control such as drainage of abscesses or removal of catheters, which may be associated with outcomes such as mortality and length of stay as well as the receipt of antimicrobial therapy[17].

## Conclusion

In conclusion, we observed that appropriate empiric antimicrobial therapy was not associated with lower in-hospital mortality or shorter post-infection length of stay after controlling for important confounding variables. These data suggest that empiric antimicrobial therapy may not be critical to patient outcomes among patients with *E. coli *or *Klebsiella *bloodstream infections. One hypothesis to explain these findings is that definitive therapy (i.e. therapy given after culture results are known) may be a more important predictor of outcomes following bacteremia than empiric therapy. Another hypothesis is that the interaction between the organism and the particular host is more critical to the outcome than the specific infection. In other words, the baseline severity of illness or the presence of comorbid disease may be more important than the actual infection and thus after controlling for these confounders, no effect is seen. While physicians have been willing to accept potential increases in antimicrobial resistance and adverse events in order to prescribe broad-spectrum empiric therapy with the hope that it will improve outcomes, this and other studies, suggest that the use of broad-spectrum empiric therapy may not lead to improved outcomes. If the benefit of broad-spectrum therapy is minimal, then a shift to narrower-spectrum therapy may provide a greater benefit to treated populations through reduced emergence of resistance and decreased adverse events. In addition, this study along with other previously published studies[8,9,15-17] provide enough equipoise to justify conducting a clinical trial to determine the actual benefit of empiric broad-spectrum antimicrobial therapy in patients with suspected Gram-negative bacteremia.

## Competing interests

The authors declare that they have no competing interests.

## Authors' contributions

KAT participated in the design of the study, performed the statistical analysis and drafted the manuscript. MLS assisted in the statistical analysis and helped draft the manuscript. RBO participated in the design of the study, assisted in the statistical analysis and helped draft the manuscript. JCM participated in the design of the study, assisted in the statistical analysis and helped draft the manuscript. JPF participated in the design of the study and helped draft the manuscript. ENP participated in the design of the study and helped draft the manuscript. ADH conceived of the study and participated in its' design and coordination, assisted in the statistical analysis and helped draft the manuscript. All authors read and approved the final manuscript.

## Pre-publication history

The pre-publication history for this paper can be accessed here:



## Supplementary Material

Additional file 1 Antimicrobial Susceptibilities of *Escherichia coli *and *Klebsiella *Bloodstream Isolates. This table includes the antimicrobial sensitivity patterns of the *Escherichia coli *and *Klebsiella *bloodstream isolates reported in this paper. Click here for file

## References

[B1] Pfaller MA, Jones RN, Doern GV, Kugler K (1998). Bacterial pathogens isolated from patients with bloodstream infection: frequencies of occurrence and antimicrobial susceptibility patterns from the SENTRY antimicrobial surveillance program (United States and Canada, 1997). Antimicrob Agents Chemother.

[B2] Hansen DS, Gottschau A, Kolmos HJ (1998). Epidemiology of Klebsiella bacteraemia: a case control study using Escherichia coli bacteraemia as control. J Hosp Infect.

[B3] Olesen B, Kolmos HJ, Orskov F, Orskov I, Gottschau A (1995). Bacteraemia due to Escherichia coli in a Danish university hospital, 1986–1990. Scand J Infect Dis.

[B4] Wisplinghoff H, Bischoff T, Tallent SM, Seifert H, Wenzel RP, Edmond MB (2004). Nosocomial bloodstream infections in US hospitals: analysis of 24,179 cases from a prospective nationwide surveillance study. Clin Infect Dis.

[B5] Marra AR, Wey SB, Castelo A, Gales AC, Cal RG, do Carmo Filho JR, Edmond MB, Pereira CA (2006). Nosocomial bloodstream infections caused by Klebsiella pneumoniae: impact of extended-spectrum beta-lactamase (ESBL) production on clinical outcome in a hospital with high ESBL prevalence. BMC Infect Dis.

[B6] Watanakunakorn C, Jura J (1991). Klebsiella bacteremia: a review of 196 episodes during a decade (1980–1989). Scand J Infect Dis.

[B7] (2004). National Nosocomial Infections Surveillance (NNIS) System Report, data summary from January 1992 through June issued October 2004. Am J Infect Control.

[B8] Metan G, Zarakolu P, Cakir B, Hascelik G, Uzun O (2005). Clinical outcomes and therapeutic options of bloodstream infections caused by extended-spectrum beta-lactamase-producing Escherichia coli. Int J Antimicrob Agents.

[B9] Du B, Long Y, Liu H, Chen D, Liu D, Xu Y, Xie X (2002). Extended-spectrum beta-lactamase-producing Escherichia coli and Klebsiella pneumoniae bloodstream infection: risk factors and clinical outcome. Intensive Care Med.

[B10] MacArthur RD, Miller M, Albertson T, Panacek E, Johnson D, Teoh L, Barchuk W (2004). Adequacy of early empiric antibiotic treatment and survival in severe sepsis: experience from the MONARCS trial. Clin Infect Dis.

[B11] Kollef MH, Sherman G, Ward S, Fraser VJ (1999). Inadequate antimicrobial treatment of infections: a risk factor for hospital mortality among critically ill patients. Chest.

[B12] Leibovici L, Shraga I, Drucker M, Konigsberger H, Samra Z, Pitlik SD (1998). The benefit of appropriate empirical antibiotic treatment in patients with bloodstream infection. J Intern Med.

[B13] Ibrahim EH, Sherman G, Ward S, Fraser VJ, Kollef MH (2000). The influence of inadequate antimicrobial treatment of bloodstream infections on patient outcomes in the ICU setting. Chest.

[B14] Harbarth S, Garbino J, Pugin J, Romand JA, Lew D, Pittet D (2003). Inappropriate initial antimicrobial therapy and its effect on survival in a clinical trial of immunomodulating therapy for severe sepsis. Am J Med.

[B15] Kang CI, Kim SH, Park WB, Lee KD, Kim HB, Kim EC, Oh MD, Choe KW (2004). Bloodstream infections due to extended-spectrum beta-lactamase-producing Escherichia coli and Klebsiella pneumoniae: risk factors for mortality and treatment outcome, with special emphasis on antimicrobial therapy. Antimicrob Agents Chemother.

[B16] Scarsi KK, Feinglass JM, Scheetz MH, Postelnick MJ, Bolon MK, Noskin GA (2006). Impact of inactive empiric antimicrobial therapy on inpatient mortality and length of stay. Antimicrob Agents Chemother.

[B17] Zaragoza R, Artero A, Camarena JJ, Sancho S, Gonzalez R, Nogueira JM (2003). The influence of inadequate empirical antimicrobial treatment on patients with bloodstream infections in an intensive care unit. Clin Microbiol Infect.

[B18] Peralta G, Sanchez MB, Garrido JC, De Benito I, Cano ME, Martinez-Martinez L, Roiz MP (2007). Impact of antibiotic resistance and of adequate empirical antibiotic treatment in the prognosis of patients with Escherichia coli bacteraemia. J Antimicrob Chemother.

[B19] Kuikka A, Sivonen A, Emelianova A, Valtonen VV (1997). Prognostic factors associated with improved outcome of Escherichia coli bacteremia in a Finnish university hospital. Eur J Clin Microbiol Infect Dis.

[B20] Osih RB, McGregor JC, Rich SE, Moore AC, Furuno JP, Perencevich EN, Harris AD (2007). Impact of empiric antibiotic therapy on outcomes in patients with Pseudomonas aeruginosa bacteremia. Antimicrob Agents Chemother.

[B21] Sunenshine RH, Wright MO, Maragakis LL, Harris AD, Song X, Hebden J, Cosgrove SE, Anderson A, Carnell J, Jernigan DB (2007). Multidrug-resistant Acinetobacter infection mortality rate and length of hospitalization. Emerg Infect Dis.

[B22] Knaus WA, Wagner DP, Draper EA, Zimmerman JE, Bergner M, Bastos PG, Sirio CA, Murphy DJ, Lotring T, Damiano A (1991). The APACHE III prognostic system. Risk prediction of hospital mortality for critically ill hospitalized adults. Chest.

[B23] Perencevich EN (2000). Excess shock and mortality in Staphylococcus aureus related to methicillin resistance. Clin Infect Dis.

[B24] Wagner DP, Knaus WA, Draper EA (1983). Statistical validation of a severity of illness measure. Am J Public Health.

[B25] Von Korff M, Wagner EH, Saunders K (1992). A chronic disease score from automated pharmacy data. J Clin Epidemiol.

[B26] Kaye KS, Sands K, Donahue JG, Chan KA, Fishman P, Platt R (2001). Preoperative drug dispensing as predictor of surgical site infection. Emerg Infect Dis.

[B27] McGregor JC, Kim PW, Perencevich EN, Bradham DD, Furuno JP, Kaye KS, Fink JC, Langenberg P, Roghmann MC, Harris AD (2005). Utility of the Chronic Disease Score and Charlson Comorbidity Index as Comorbidity Measures for Use in Epidemiologic Studies of Antibiotic-resistant Organisms. Am J Epidemiol.

[B28] McGregor JC, Rich SE, Harris AD, Perencevich EN, Osih R, Lodise TP, Miller RR, Furuno JP (2007). A systematic review of the methods used to assess the association between appropriate antibiotic therapy and mortality in bacteremic patients. Clin Infect Dis.

[B29] Hamilton KW, Bilker WB, Lautenbach E (2007). Controlling for severity of illness in assessment of the association between antimicrobial-resistant infection and mortality: impact of calculation of Acute Physiology and Chronic Health Evaluation (APACHE) II scores at different time points. Infect Control Hosp Epidemiol.

