# Supplementary material for: Impact of Empiric Antimicrobial Therapy on Outcomes in Patients with Escherichia coli and Klebsiella pneumoniae Bacteremia: A Cohort Study
Source: BMC Infect Dis. 2008 Sep 15;8:116. doi: 10.1186/1471-2334-8-116 (PMC2551598; doi:10.1186/1471-2334-8-116)
Supplement: Additional file 1 — Antimicrobial Susceptibilities of Escherichia coli and Klebsiella Bloodstream Isolates. This table includes the antimicrobial sensitivity patterns of the Escherichia coli and Klebsiella bloodstream isolates reported in this paper. [file 1471-2334-8-116-S1.doc]

| **Antimicrobial Susceptibilities of Escherichia coli and Klebsiella Bloodstream Isolates**  **(Listed as percent of isolates susceptible*)** | | | | | | | | | | |
| --- | --- | --- | --- | --- | --- | --- | --- | --- | --- | --- |
| **Isolates** | **No. of Isolates** | **Aminoglycosides** | **Beta-Lactams** | | | **Cephalosporins** | | | **Quinolones**** | **Sulfa** |
|  | Total, N = 416 | Gentamicin | Ampicillin-Sulbactam | Pipericillin-Tazobactam | Imipenem | Cefazolin | Cefepime | Ceftriaxone | Ciprofloxacin  Levofloxacin  Gatifloxacin | Trimethoprim-Sulfa |
| *Escherichia coli* | 225 (54%) | 83% (186/224) | 67% (141/211) | 93% (209/225) | 98% (170/173) | 83% (184/222) | 93% (208/224) | 92% (206/223) | 71% (158/224) | 61% (138/225) |
| *Klebsiella* species | 203 (49%) | 92% (186/203) | 69% (137/199) | 83% (169/203) | 97% (172/177) | 76% (152/201) | 91% (182/201) | 86% (171/200) | 88% (179/203) | 70% (143/202) |

*Listed as percent of isolates susceptible according to CLSI definitions

** Susceptibility testing for quinolones was performed using one of the following: ciprofloxacin, levofloxaxin or gatifloxacin
